# How does the general public view posthumous organ donation? A meta-synthesis of the qualitative literature

**DOI:** 10.1186/1471-2458-11-791

**Published:** 2011-10-11

**Authors:** Joshua D Newton

**Affiliations:** 1 Department of Marketing, Monash University, Caulfield, Victoria, Australia; 2 School of Psychology and Psychiatry, Monash University, Caulfield, Victoria, Australia

## Abstract

**Background:**

Many individuals are unwilling to become posthumous organ donors, resulting in a disparity between the supply and demand for organ transplants. A meta-synthesis of the qualitative literature was therefore conducted to determine how the general public views posthumous organ donation.

**Methods:**

Three online databases (PubMed, PsycINFO, Scopus) were searched for articles published between January 1990 and May 2008 using the following search terms: organ donation, qualitative, interview. Eligibility criteria were: examination of beliefs about posthumous organ donation; utilization of a qualitative research design; and publication in an English peer-reviewed journal. Exclusion criteria were examining how health professionals or family members of organ donors viewed posthumous organ donation. Grounded theory was used to identify the beliefs emerging from this literature. Thematically-related beliefs were then grouped to form themes.

**Results:**

27 articles from 24 studies met the inclusion criteria and were reviewed. The major themes identified were: religion, death, altruism, personal relevance, the body, the family, medical professionals, and transplant recipients. An altruistic motivation to help others emerged as the most commonly identified motivator for becoming an organ donor, although feeling a sense of solidarity with the broader community and believing that donated organs are put to good use may be important preconditions for the emergence of this motivation. The two most commonly identified barriers were the need to maintain bodily integrity to safeguard progression into the afterlife and the unethical recovery of organs by medical professionals. The influence of stakeholder groups on willingness to become an organ donor was also found to vary by the level of control that each stakeholder group exerted over the donation recovery process and their perceived conflict of interest in wanting organ donation to proceed.

**Conclusions:**

These findings afford insights into how individuals perceive posthumous organ donation.

## Background

Demand for transplantable organs currently exceeds supply [1], and one of the principle reasons for this disparity is that many next of kin are reluctant to allow organ donation to proceed. In the United States of America, for example, next of kin withhold consent for organ donation in 47% of eligible cases [2], and similar rates of refusal have been reported in both Australia [3] and the United Kingdom [4]. However, next of kin are more likely to grant permission for organ recovery if the deceased had indicated during their lifetime a willingness to become an organ donor [5,6]. Understanding the beliefs that encourage or dissuade individuals from becoming willing organ donors is therefore of particular importance.

Early efforts to examine the factors that influence donation willingness made use of quantitative research designs [7-10]. While such designs are well suited to enumerating the beliefs that influence behavior, they are less amenable to understanding how individuals perceive, conceptualize, and give meaning to issues such as organ donation [11]. To redress this limitation, qualitative methods have more recently been used to complement the results obtained by quantitative studies. One of the strengths of qualitative designs is that they allow researchers to explore the multifaceted, inter-related, private, and often conflicting beliefs held by individuals [11]. Qualitative designs also allow researchers to clarify what individuals actually mean when they describe particular beliefs or experiences [11]. Qualitative research can therefore provide unique insights into how individuals perceive organ donation. Unfortunately, much of the qualitative research on organ donation has been conducted with small samples or among populations with unique characteristics (see Table 1). As a result, findings from qualitative studies are often of limited generalizability.

**Table 1:** Characteristics of the Studies Reviewed in the Meta-synthesis

Study	Reference	Country	Participants	Recruitment	Data collection
1	Albright et al., 2005 [13]	USA	57 Filipino adolescents & adults	Purposive sampling	Focus groups
2	AlKhawari et al., 2005 [14]	UK	141 Indo-Asian adults	Convenience sampling through Islamic centers	Semi-structured interviews & focus groups
3	Arriola et al., 2005 [15]; Arriola et al., 2007 [16]	USA	68 African-American adults	Convenience sampling through Churches	Focus groups
4	Bhengu & Uys, 2004 [17]	South Africa	1 non-Zulu speaking & 47 Zulu speaking adults	Purposive & snowball sampling	Semi-structured interviews
5	Braun & Nichols, 1997 [18]	USA	7 Chinese American adults, 8 Japanese American adults, 10 Vietnamese American adults, & 11 Filipino American adults	Purposive & snowball sampling	Semi-structured interviews & focus groups
6	Davis & Randhawa, 2004 [19]; Davis & Randhawa, 2006 [20]	UK	120 African & Caribbean adults	Purposive sampling	Focus groups
7	Exley et al., 1996 [21]	UK	22 Sikh adults	Purposive sampling	Semi-structured interviews & focus groups
8	Fahrenwald & Stabnow, 2005 [22]	USA	21 Oglala Lakota Sioux adults	Snowball sampling	Semi-structured interviews
9	Frates & Garcia Bohrer, 2002 [23]	USA	22 Hispanic adults	Telephone and purposive sampling	Semi-structured interviews
10	Hayward & Madill, 2003 [24]	UK	10 Pakistani & 17 white English adults	Convenience & snowball sampling	Focus groups
11	Kennedy, 2002 [25]	India	6 English-speaking adults^†^	Convenience sampling	Discursive interviews
12	Lai et al., 2007 [26]	UK	14 adult women from the general population	Snowball sampling	Active interviews
13	Moloney & Walker, 2002 [27]	Australia	29 adults from the general population	Randomly selected from electoral rolls	Focus groups
14	Molzahn et al., 2004 [28]	Canada	14 Coast Salish adults	Purposive & snowball sampling	Semi-structured interviews
15	Molzahn et al., 2005 [29]	Canada	39 Chinese adults	Purposive & snowball sampling	Focus groups & semi-structured interviews
16	Molzahn et al., 2005 [30]	Canada	40 South Asian adults	Purposive & snowball sampling	Focus groups & semi-structured interviews
17	Morgan, Mayblin et al., 2008 [31]	UK	14 Caribbean adults	Convenience & snow-ball sampling	Semi-structured interviews
18	Morgan, Harrison et al., 2008 [32]; Morgan et al., 2005 [33]	USA	78 family-pair dyads (156 adults) from the general population	Advertisements	Observation of communication between dyads
19	Peters et al., 1996 [34]	USA	51 registered donors & 51 non-registered donors from the general population	Not specified	Focus groups
20	Randhawa, 1998 [35]	UK	16 Sikh, 32 Muslim & 16 Hindu adults, all originally from South Asia	Randomly selected from electoral rolls	Focus groups
21	Sanner, 1994 [36]	Sweden	38 adults from the general population	Purposive sampling	Semi-structured interviews
22	Sanner, 2001 [37]	Sweden	69 adults from the general population	Purposive sampling	Semi-structured interviews
23	Thompson, 1993 [38]	USA	30 African-American adolescents & 26 African-American adults	Convenience sampling	Focus groups
24	Wittig, 2001 [39]	USA	10 African-American women	Purposive sampling	Semi-structured interviews

^† ^Comments from two doctors, which were analyzed separately in this article, were not examined.

One way of addressing the limited generalizability of qualitative research is to conduct a meta-synthesis of the available research [12]. In a meta-synthesis, findings from multiple qualitative studies are aggregated so that the factors that shape social phenomena across multiple populations can be identified [12]. In the context of organ donation, such an analysis would provide clinicians, policy makers, and researchers with a deeper understanding of the beliefs that encourage or dissuade individuals from becoming willing organ donors. A meta-synthesis of the qualitative research on organ donation has not, however, been previously conducted. The current study was therefore designed to address this gap.

## Methods

### Study selection

**Figure 1:**
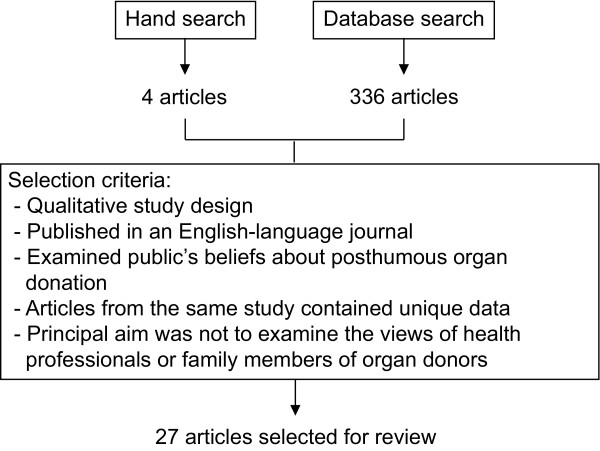
**Literature search strategy**. Figure 1 illustrates the number of articles that were identified through database searches and a hand search of article reference lists. The inclusion criteria used to select the final 27 articles for review are also presented.

Three electronic bibliographic databases (PubMed, PsycINFO, Scopus) were searched using the following terms: "organ donation AND interview" and "organ donation AND qualitative". These search terms were applied to articles published between January 1^st ^1990 and May 31^st ^2008. Articles were also found by hand-searching the reference lists of relevant articles. Articles identified through this process were selected for analysis if they: (i) utilized a qualitative study design; (ii) were published in an English-language, peer-reviewed journal; and (iii) investigated the beliefs that individuals hold about posthumous organ donation. Studies were excluded if their primary aim was to examine: (i) how individuals arrive at a decision about donating the organs of a deceased family member; or (ii) the reactions of health professionals to posthumous organ donation. Multiple papers from a single study were included for analysis if each paper presented unique data. A summary of the search strategy and selection criteria utilized in the current study is presented in Figure 1. Twenty-seven articles from 24 studies [13-39] satisfied the selection criteria and were included in the review (see Table 1). These studies represented the views of 1,213 participants towards organ donation.

### Analysis

Qualitative data extracted from the results section of each reviewed study were analyzed using a grounded theory framework [40], a method of analysis that has also been used in other meta-syntheses [41,42]. Following a grounded theory methodology, data about individuals' perceptions of organ donation were inductively coded to create a series of belief-based concepts. Constant comparisons between and within belief concepts were undertaken throughout this process to ensure that each concept provided an accurate representation of the original qualitative data. Similar concepts were then grouped to form thematic categories. To enhance methodological rigor, a second coder independently analyzed the data using the belief-based concepts generated by the first coder. Feedback from the second coder also led to several minor changes being made to the belief-based concepts. The coding schemes from the first and second coders were then compared, and any coding inconsistencies were resolved through discussion.

## Results

Seven key thematic categories that impacted on individuals' willingness to become an organ donor were identified. Each theme encapsulated a series of positive or negative beliefs about organ donation (see Table 2).

**Table 2:** Beliefs Identified in the Reviewed Studies

Beliefs	**Study**^†^																								*n*
		
	1	2	3	4	5	6	7	8	9	10	11	12	13	14	15	16	17	18	19	20	21	22	23	24	
*Religion*																									
Religious opposition to organ donation	+	+	+	+	+	+	+	+	+	+	+	+	+	+	+	+		+	+	+	+	+	+	+	23
Need to maintain bodily integrity	+	+	+	+	+	+	+	+	+	+	+	+	+	+	+			+	+	+	+	+	+	+	22
Interferes with funeral or burial rituals		+		+	+			+		+			+	+	+	+		+		+	+				12
Fatalism		+		+		+				+				+		+					+			+	8
Haunt surviving family members				+		+																			2
Religious support for organ donation	+		+	+	+		+				+				+	+		+		+					10
Uncertain of religion's position		+	+	+	+		+			+	+				+	+		+	+	+			+		13
																									
*Death*																									
Don't like to think/talk about death or organ donation	+	+		+	+	+	+		+	+	+	+		+	+	+		+				+	+	+	17
Talking about death or organ donation tempts fate				+		+			+		+					+		+					+	+	8
Organ donation transforms the concept of death		+							+				+					+					+	+	6
																									
*Altruism*																									
Organ donation helps those in need	+	+	+		+		+	+	+	+	+	+	+	+	+	+	+	+	+	+	+			+	20
Organ donation helps the broader community			+					+		+			+	+				+							6
Indirect reciprocity			+	+									+					+			+				5
Social/cultural isolation		+				+											+								3
																									
*Personal relevance*																									
Don't consider donation until its personally relevant	+	+			+	+				+				+		+		+						+	9
Beliefs change as donation becomes personally relevant	+		+			+		+		+	+		+											+	8
																									
*The body*																									
Organ donation dehumanizes the body	+	+	+		+		+	+		+			+			+	+	+		+	+				13
Ownership over body	+									+		+					+			+	+	+			7
Some organs have special significance				+	+					+		+			+					+	+	+			8
Personality contamination				+						+				+				+		+		+			6
Utilitarian view of the body			+		+			+		+			+					+		+	+	+		+	10
Body holds little intrinsic importance after death			+		+			+					+					+			+			+	7
Wasteful not to donate					+													+		+					3
Body is a 'machine'										+		+	+								+	+			5
																									
*The family*																									
Family attitude towards donation	+	+		+		+	+	+		+	+				+	+		+						+	12
Minimize family stress		+						+		+			+	+						+	+				7
																									
*The medical profession*																									
Organs obtained unethically	+	+	+	+	+	+	+		+	+			+	+	+	+	+	+	+	+	+				18
Organs removed before death	+	+					+						+	+	+			+		+	+				9
Life-saving medical care withheld		+	+	+	+	+	+		+	+			+			+	+	+	+						13
Exceed terms of consent				+		+											+	+			+				5
Doctors make mistakes			+	+			+		+				+				+	+			+				8
Brain death				+			+		+				+				+	+			+				7
Routine medical errors			+															+							2
Life needlessly prolonged to obtain organs						+															+				2
Doctors push boundaries of nature																		+			+	+	+		4
Unethical or prejudicial organ allocation		+	+			+	+			+			+					+	+		+		+		10
Organs used for unintended purposes							+		+		+		+			+		+			+				7
Illicit trading							+		+		+		+			+		+							6
Unauthorized experiments																					+				1
Health system constraints				+				+		+													+		4
Unaware of registration process						+			+					+	+	+									5
Perceived lack of donation knowledge	+		+	+		+		+	+		+							+		+			+		10
																									
*The transplant recipients*																									
Donated organ may be defective	+							+		+			+			+		+		+	+	+		+	10
Recipients 'waiting' for someone to die													+												1
Organ allocation should be restricted to:		+	+	+		+				+	+			+	+	+		+				+		+	12
Specific populations		+	+							+	+			+											5
'Deserving' recipients		+		+						+								+							4
Family or friends			+	+		+				+				+	+	+						+		+	9

^† ^Numbers correspond to the studies summarized in Table 1.

### Theme 1: Religion

Religious beliefs about organ donation existed along a continuum that ranged from outright rejection of organ donation to unequivocal support. The religious belief most commonly used to reject organ donation was the notion that bodily integrity should be maintained to safeguard progression into the afterlife.

"The organs will be witness to your actions on Judgement Day." *UK, Indo-Asian, Muslim, male, 37 years old *[14]

This belief was by no means universal among those who shared the same faith, however, suggesting that many religious beliefs are personalized interpretations of more general religious precepts.

"When we die it's not our body that goes, it's our soul that goes." *UK, Pakistani, Muslim, female *[24]

A second religious belief perceived as being in opposition to organ donation was concern that organ retrieval could prevent the observance of specific funeral or burial rituals.

"With us, only close relatives, no more than four, should touch the body (to wash it) and place it in the grave." *UK, South Asian, Muslim *[35]

Fatalism, or the notion that all events are predestined, also shaped views about organ donation. Fatalistic individuals typically spoke about organ transplantation as being either unnecessary or against the will of a divine being. As a consequence, they opposed the need for both organ donation and transplantation.

"You die when the Creator thinks it's time for you to die, not to extend a person's life simply because of their age, or, you know, because there is a chance to do so." *Canada, Coast Salish *[28]

At the other end of the continuum were religious beliefs that were seen as providing support for organ donation. While many of the religious barriers to organ donation were very specific in nature, religious support for becoming an organ donor was derived from more general precepts about the importance of helping others.

"It's better to give than to receive." *Canada, Indian, Christian *[30]

"If you save the one life, then you save the whole world." *Canada, Indian, Muslim *[30]

Although many individuals believed their religion either supported or opposed organ donation, some expressed uncertainty about their religion's stance towards organ donation. One of the reasons for this uncertainty was the perception that religious texts provide little instruction concerning the appropriateness of organ donation. These individuals therefore wished to clarify their religion's position before they made a decision about organ donation.

"I do not know anything myself, but I would ask the scholars... If they tell us it is alright, then it is alright ... If they say no, that is no." *UK, Indo-Asian, Muslim, male, 38 years old *[14]

### Theme 2: Death

Concerns about death intersected with beliefs about organ donation. Those who feared thinking or talking about death, for instance, were often reluctant to consider the issue of organ donation. Declaring one's donation wishes was also seen to be tempting fate.

"Buying a lot in the cemetery, they [would] rather not talk about it. So if you raise the question about donating your parts after you die, they probably don't want to hear about it." *Canada, Chinese *[29]

"Talking about bad things sometime gives them the power to happen." *USA, African-American, female *[39]

Others, in contrast, saw organ donation as a way to transcend the finality of death and achieve symbolic immortality by allowing the donor to 'live on' in the transplant recipient.

"You often hear people say, well my child, my brother gave something to another person, so therefore they live on." *Australia, male *[27]

### Theme 3: Altruism

Willingness to become an organ donor was often expressed in terms of an altruistic, non-religiously-motivated desire to help individuals in need.

"When you give an organ, you're doing it to help somebody." *USA, African-American *[15]

Alternative altruistic motivations for becoming an organ donor also emerged. Some respondents, for instance, situated their desire to become an organ donor within the broader context of helping the general community.

"I am also in favour [of organ donation] because it is a service to humanity and it is giving life ... because you are, of course, dying so it is better off that you save someone else's life, like a gift for society." *Australia, male *[27]

Others justified their support for organ donation by making reference to indirect reciprocity. Indirect reciprocity refers to the notion that an individual is duty bound to help others as they themselves would want to be helped.

"I'm sure if you needed an organ you would want someone to donate if it was going to save your life. You would want somebody to donate." *USA, African-American *[15]

Identifying with the broader community may, however, be an important precondition for the expression of these altruistic ideals. Specifically, some respondents who felt socially or culturally isolated indicated an unwillingness to donate their organs.

"It's like, you're black first, a man second. That stops you feeling part of it." *UK, Caribbean, male *[31]

### Theme 4: Personal relevance

Personal relevance appeared to play an important role in how respondents interacted with the notion of organ donation. For many, consideration of organ donation did not take place until it became contextualized within the lived experience of an acquaintance or family member.

"It didn't dawn on us to discuss it [organ donation] unless, of course, there's a situation - when it's close to home. But if you are comfortable right now, it seldom happens." *USA, Filipino *[13]

Personal relevance also appeared to act as a catalyst for broader attitude change. One respondent, for instance, described the experience of watching a close friend's daughter receive dialysis while waiting for an organ transplant.

"I had an aversion to donation, but with the sort of awareness I have now, it is important that everyone, everyone carries a donor card." *UK, African, male, 31-44 years old *[19]

### Theme 5: The body

The body was an integral component of many individuals' self-identity. As such, actions that could dehumanize or threaten the dignity or individuality of the body were viewed with suspicion and distress. Examples of such beliefs included the notion that organ donation could lead to the body being treated as a collection of 'spare parts' or as cuts of meat in a butchers shop.

"Thinking of them as a hunk of meat, like a piece of sheep or something. That is how doctors think." *Australia, male *[27]

"The human body is not a machine. Organ transplantation is abusing the body's dignity. Our organs are not mere spare parts. They are a gift from Allah." *UK, Indo-Asian, male, 24 years old *[14]

As a result, the prospect of organ donation often fuelled a desire to retain control over the body, even after death.

"It (the body) is your possession. The more you use it, the more you value it, the more prize possession it becomes, the less you want it harmed, or taken apart, or damaged or illness to come to you." *UK, female, 21 years old *[26]

Nevertheless, there was some evidence to suggest that such concerns may be resolved by reassuring individuals that organ recovery is carried out respectfully and in a manner that preserves the outward appearance of the body.

"I can donate everything just as long I look OK in my coffin." *USA, Filipino *[13]

While some individuals rejected organ donation out of a desire to preserve the dignity of the whole body, others felt that organ recovery could take place so long as certain parts of the body were left untouched. The eyes and heart, for instance, were sometimes thought to hold particular significance because of the perceived centrality of these parts of the body to an individual's sense of self.

"I don't want to give my heart and my eyes because I feel like you think things with your heart." *UK, white, female *[24]

An associated belief was the notion that aspects of an individual's personality are situated within specific body parts. Individuals who held this belief therefore feared that organ transplantation could result in personality contamination.

"I don't like the idea of my organs living in another body, it may affect their personality and make them more like me." *UK, Pakistani, female *[35]

Other individuals held more utilitarian views of the body, believing that the body gave physical form to the self but was not an integral component of their self-identity. For these individuals, the body held little intrinsic importance after death.

"Who cares what happens to my body ... after I die. I don't care one way or the other. If there's someone who needs my organs, sure, why not, my soul will be gone anyway." *USA, Oglala Lakota Sioux *[22]

As a result, refusing to become an organ donor was deemed to be a waste of healthy and potentially life-saving organs.

"I'd rather not go to waste if someone could use it." *USA *[32]

One way that individuals explained this utilitarian view to others was by equating organ transplantation with the replacement of faulty parts in a machine. As outlined earlier, this view was seen by others as dehumanizing the body.

"Cynically speaking, it's machine parts, in principle nothing strange." *Sweden *[37]

### Theme 6: The family

Individuals appeared desirous of maintaining family cohesion and stability and were consequently influenced by the donation attitudes held by their family members.

"I personally have no objection but my father does, so I am not sure." *UK, Indo-Asian, female, 20 years old *[14]

Further indicative of this desire to maintain family stability was an inclination to minimize stress among surviving family members. For some, this meant making their donation wishes known to family members in order to take:

"The emotive onus away from your relatives." *UK, white, female *[24]

For others, it meant refusing to become an organ donor so that their families would not have to see their body following organ recovery.

"I don't like the idea of my relatives having to see my body having been carved up." *UK, Gujarati, male *[35]

### Theme 7: The medical profession

Mistrust of the medical profession was a common cause of anxiety across the reviewed papers. The most common variant of this concern was that organs would be recovered unethically. Some, for instance, feared that doctors would deliberately remove a patient's organs before the patient had died, while others believed that life-saving medical care would be withheld so that patients could become eligible for organ donation. There was also a belief that medical staff go beyond an individual's terms of consent to obtain additional organs.

"If I sign up to donate a kidney, they may take out everything else as well." *UK, Caribbean, male, 31-44 years old *[19]

While some believed that organs may be unethically procured, others thought that organs could be taken prematurely as a result of mistakes made during organ recovery. The concept of brain death was particularly singled out as a potential barrier to becoming a willing donor, with questions being raised about doctors' ability to determine if a patient met the criteria for brain death or whether the medical profession more generally was mistaken for using brain death as the basis for allowing donation to proceed.

"Brain death may not be definitive so if you do donate your organs you've given away your (or someone else's) last chance at life." *Australia *[27]

Fears about more routine medical errors were also identified.

"The medical team, although they admitted making the mistake, made that mistake ... that's really going to, uh, cause some thinking now before anyone wants to donate or receive an organ." *USA, African-American *[15]

Juxtaposed with the notion that organs are removed prematurely was the belief that doctors prolong life unnecessarily to obtain organs. There was also concern that the medical profession was too eager to push the boundaries of nature.

"The desire to play that many doctors have is really dangerous. In the end they will breach the borders that nature has determined." *Sweden *[37]

Other medical-related beliefs about organ donation also emerged. One concern was that the process of allocating organs was biased, particularly against ethnic minorities or the poor.

"I still think it's a racial thing too because most Black people feel that they're never going to get an organ anyway because the organs are going to go to those people with money, or those people who know someone, or White people." *USA, African-American *[16]

There was also a fear that organs could be used for unintended purposes, violating the altruistic reasons for which these organs had been donated. These unintended purposes included the illicit trading of organs, especially for monetary gain, and the use of donated organs in unauthorized medical experiments.

Perceived knowledge gaps, either about organ recovery or the method for recording donation wishes, were also identified as barriers to becoming an organ donor. Likewise, health system constraints were seen as reducing the impetus to record donation wishes. An individual living on a reservation in the United States of America, for instance, questioned whether health professionals at the local hospital examined drivers' licenses to identify the donation wishes of the deceased.

"If you die up here they don't look at those things. They don't look at your driver's license. They just send you to the mortuary." *USA, Oglala Lakota Sioux *[22]

### Theme 8: The recipients

Concerns about the potential recipients of donated organs were often identified during data analysis. These concerns were sometimes motivated by a wish to safeguard potential transplant recipients. Several individuals, for instance, were reluctant to become organ donors because they feared donating a defective organ.

"I want to be a donor. I don't think they would want any of my trash. Who wants to sell ugly arteries?" *USA, Oglala Lakota Sioux *[22]

More often, however, concerns centered on the attitudes or suitability of potential recipients. Some, for instance, believed that potential transplant recipients were 'willing' others to die.

"All these people who might have bad hearts or whatever may be sitting in the hospital praying God why can't somebody crash into a tree and a nice 17 year old would do me fine." *Australia, female *[27]

Others wanted to restrict organ allocation to individuals that satisfied particular criteria, such as members of a specific community, 'deserving' recipients, or family and friends. Motivations for wanting to restrict allocation varied but included remedying perceived injustices in organ allocation and ensuring that the lifesaving gift of organ transplantation was only received by those deemed worthy of such a gift.

"I wouldn't mind if my organs were donated to a black person or minority person, but what are those chances that they would get them? You know because blacks are always put down ... we're always on the bottom of the list as far as organ donors." *USA, African American *[15]

"I want to make sure that the person is good before I give him my organs." *UK, Indo-Asian, male, 20 years old *[14]

## Discussion

A qualitative meta-synthesis was used to identify a comprehensive set of community beliefs about posthumous organ donation. A striking finding that emerged from this analytical approach was the identification of thematically similar beliefs across culturally diverse study samples. The notion that organ donation interferes with funeral or burial rituals, for example, was identified in samples of: ethnic Asians living in Canada [29,30], the United Kingdom [14,24,35], and the United States of America [18]; the indigenous peoples of Canada [28] and the United States of America [22]; Zulu-speaking adults living in South Africa [17]; and individuals drawn from the general American [32], Australian [27], and Swedish [36] populations. These findings attest to the ubiquitous nature of many beliefs about organ donation and suggest that public health campaigns developed in one cultural context may also have relevance in other contexts.

The most commonly identified motivator for becoming an organ donor was a desire to help others in need, highlighting the importance of altruistic motivations in the decision to become an organ donor. Nevertheless, several boundary conditions for the emergence of such altruistic wishes were identified. Those who felt socially alienated, for instance, were often unwilling to contribute to a community in which they felt no part. More generally, factors that could limit the beneficial outcomes of their altruistic actions, such as perceived injustices in organ allocation or the use of donated organs for unauthorized experiments or monetary gain, appeared to reduce individuals' impetus to become willing organ donors. Thus, for many individuals, feeling a sense of solidarity with the wider community and perceiving that donated organs are put to good use may be necessary preconditions for becoming a willing organ donor.

The two most commonly identified barriers to becoming a willing organ donor were: (i) the need to maintain bodily integrity to safeguard progression into the afterlife; and (ii) the unethical recovery of organs by medical professionals. Both beliefs indicated a desire to avoid death, either in a spiritual or a physical sense. Nevertheless, both beliefs reflect an apparent lack of awareness regarding the widespread interfaith support for organ donation [43,44] and the strict procedural checks and balances that have been instituted to safeguard the probity of the organ recovery process [45].

The belief that organs are recovered unethically was indicative of a more general feeling of mistrust about the role of the medical profession in organ donation. This mistrust appeared to stem from two issues. First, medical professionals were seen to have direct control over the recovery and subsequent transplantation of donated organs. Second, medical professionals had a perceived conflict of interest in that although they are professionally obligated to help potential transplant recipients, they are also responsible for treating organ donors. Thus, doctors were perceived to have both the means and the motive for performing unethical organ recovery.

**Figure 2:**
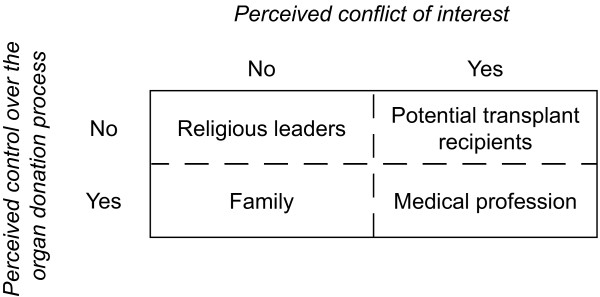
**Perceived conflict of interest and control**. Figure 2 outlines the perceived levels of conflict of interest and control for each of the key stakeholders involved in the organ donation process.

The dimensions of control and conflict of interest could also explain why potential transplant recipients, family members, and religious leaders engendered different responses from members of the general community (see Figure 2). Potential transplant recipients, for instance, elicited either revulsion (e.g., potential recipients 'willing' individuals to become organ donors) or altruism (e.g., potential recipients at the mercy of others for the alleviation of their ill-health) because they exerted no control over the organ donation process but had a perceived conflict of interest in wanting donation to proceed. In contrast, individuals often expressed a desire to reduce the emotional strain of organ donation on their family because family members had no perceived conflict of interest in wanting organ donation to take place but played a fundamental role in determining whether donation could proceed. Finally, religious leaders were seen as a trustworthy source for clarifying the religious precepts associated with organ donation because they had neither control over organ recovery nor a conflict of interest in promoting organ donation. Researchers, policy makers, and medical professionals interested in promoting organ donation should therefore be mindful of how perceptions of the key stakeholders involved in the organ donation process can influence individuals' willingness to become an organ donor.

Several limitations were associated with the current study. One limitation was that certain groups, such as adolescents and individuals from developing economies, were underrepresented in the reviewed studies. Additional research is therefore required to determine whether the donation beliefs identified in the current study are also held by individuals within these cohorts. A second limitation was that analysis could only be conducted on the qualitative information that authors had included in their articles. There is consequently a risk that important beliefs may not have been available for analysis. Nevertheless, the large number of studies included in the current review goes some way towards reducing this risk.

A third limitation was that the meta-synthesis could not evaluate the relative influence of the identified beliefs on organ donation-related behaviors. Unfortunately, estimating the relative influence of these beliefs from the existing quantitative literature is difficult, for the quantitative literature has not typically examined beliefs about organ donation with the same level of specificity as was attained in the current study. Nevertheless, estimates of behavioral influence can be made for some of the thematic categories by examining the results of quantitative studies that have assessed conceptually similar constructs. Recent meta-analytic findings [46], for instance, suggest that social referents (pooled effect size (ES) = 1.89) have the strongest positive effect on organ donor status, followed by religion (pooled ES = 1.64), knowledge about organ donation (pooled ES = 1.39), and altruism (pooled ES = 1.23). In contrast, fear of organ donation (pooled ES = 0.41) has the strongest negative effect on organ donor status, followed by fear of death (pooled ES = 0.62). As such, in the absence of fine-grained belief-based data, pooled effect sizes from meta-analytic studies may provide useful insights into the relative importance of the thematic categories identified in this study.

## Conclusions

The findings from the current study provide a general set of beliefs that members of the general public could potentially hold about organ donation. These beliefs could be used to develop quantitative measures aimed at identifying how specific populations perceive organ donation. The identified beliefs may also be useful to policymakers and researchers interested in promoting organ donation through public health campaigns and other community-level initiatives.

## Competing interests

The authors declare that they have no competing interests.

## Authors' contributions

JN conceived of the study, conducted the literature search and article coding, and drafted the manuscript.

## Pre-publication history

The pre-publication history for this paper can be accessed here:

http://www.biomedcentral.com/1471-2458/11/791/prepub
